# Combined Effects of Energy Development and Disease on Greater Sage-Grouse

**DOI:** 10.1371/journal.pone.0071256

**Published:** 2013-08-05

**Authors:** Rebecca L. Taylor, Jason D. Tack, David E. Naugle, L. Scott Mills

**Affiliations:** Wildlife Biology Program, University of Montana, Missoula, Montana, United States of America; University of Georgia, United States of America

## Abstract

Species of conservation concern are increasingly threatened by multiple, anthropogenic stressors which are outside their evolutionary experience. Greater sage-grouse are highly susceptible to the impacts of two such stressors: oil and gas (energy) development and West Nile virus (WNv). However, the combined effects of these stressors and their potential interactions have not been quantified. We used lek (breeding ground) counts across a landscape encompassing extensive local and regional variation in the intensity of energy development to quantify the effects of energy development on lek counts, in years with widespread WNv outbreaks and in years without widespread outbreaks. We then predicted the effects of well density and WNv outbreak years on sage-grouse in northeast Wyoming. Absent an outbreak year, drilling an undeveloped landscape to a high permitting level (3.1 wells/km^2^) resulted in a 61% reduction in the total number of males counted in northeast Wyoming (total count). This was similar in magnitude to the 55% total count reduction that resulted from an outbreak year alone. However, energy-associated reductions in the total count resulted from a decrease in the mean count at active leks, whereas outbreak-associated reductions resulted from a near doubling of the lek inactivity rate (proportion of leks with a last count = 0). Lek inactivity quadrupled when 3.1 wells/km^2^ was combined with an outbreak year, compared to no energy development and no outbreak. Conservation measures should maintain sagebrush landscapes large and intact enough so that leks are not chronically reduced in size due to energy development, and therefore vulnerable to becoming inactive due to additional stressors.

## Introduction

Population response to multiple stressors is among the top ten priorities for science to inform conservation and management policy in the United States [1]. Multiple stressors precipitate the extinction of species [2], and limited understanding of cumulative and interactive effects hamper assessments required by the National Environmental Policy Act and the Endangered Species Act [3], [1]. Oil and gas (energy) development is an ongoing stressor to wildlife populations on lands throughout the western United States [4], and in 2002, West Nile virus (WNv) emerged as an additional stressor to these populations [5]. The greater sage-grouse (*Centrocercus urophasianus*; sage-grouse) in northeast Wyoming provides a case study demonstrating the potential consequences of multiple stressors, such as energy development and disease, on a species of conservation interest.

The sage-grouse is a sagebrush (*Artemisia spp.*) obligate species [6], and habitat loss and degradation has resulted in a ≥4 decade long population decline [7], [8]. The species currently occurs in 11 western states and occupies ≤54% of its pre-European settlement range [9]. Wyoming provides habitat for nearly 40% of the range-wide population [10], and landscapes being developed for energy extraction in Wyoming contain some of the highest sage-grouse abundances in North America [11]. Breeding sage-grouse populations are severely impacted at commonly permitted oil and gas well densities [12]. Impacts have been low or indiscernible at the lowest level (0.4 wells/km^2^), but at medium and high levels (1.5 and 3.1 wells/km^2^), losses on breeding grounds (leks) have been 2–5 times greater inside than outside of development, and number of males counted at remaining leks declined by 32 to 77% [13]. West Nile virus is a known stressor which may compound the impacts from oil and gas development [14]. Severe WNv outbreaks have been observed in Wyoming's sage-grouse populations since 2003 [15], and mortality from the disease could reduce population growth by an average of 6–9% per year [14]. In response to sage-grouse population declines in Wyoming, conservation areas (termed ‘core areas’) in which new energy development is restricted were delineated in 2008 [16].

While negative impacts of energy development on sage-grouse populations have been demonstrated in multiple study areas via demographic studies and counts of males on leks [12], research in areas of concentrated development [17], [18], [19] has detected impacts at smaller spatial extents than have regional studies [20], [21]. In addition, anthropogenic stressors such as energy development have been hypothesized to interact with WNv to exacerbate sage-grouse population declines [14]; however, this hypothesis has yet to be tested.

Reasons for differing estimates of the scale at which energy development impacts sage-grouse, and the untested interaction between energy and disease impacts likely stem from similar challenges in estimating stressor effects. Stressor studies are usually first conducted on a relatively small scale, at sites close to development. A small scale study near development provides a natural starting point, as the study is focused on the source of the impact, and the small spatial scale affords researchers some control over confounding factors, such as vegetation and landscape, that may mask a response to development. However, if the entire study area is close to development, effects at impacted sites may appear artificially small because the nominal control areas may also be affected by development [22]. A small scale study close to development becomes particularly problematic when multiple stressors and their potential interactions are considered. Correctly quantifying multiple main effects and interactions requires more than data with a wide range of intensities for each stressor. It also requires that the different intensities for any one stressor be observed in combination with the different intensities of each of the other stressors [23].

The goals of our research were threefold. First, we aimed to identify the scale of energy impacts to leks, using data that encompassed both local and regional variation in levels of energy development. Second, we developed a model and tested for interactions between energy development and WNv outbreak years, again accounting for both local and regional variation in levels of energy development. Third, we used our model to predict the effects of energy development on sage-grouse in the presence and absence of a WNv outbreak year in a heavily developed area of Wyoming, where energy development is an ongoing threat to sage-grouse populations.

## Methods

### Study region

**Figure 1:**
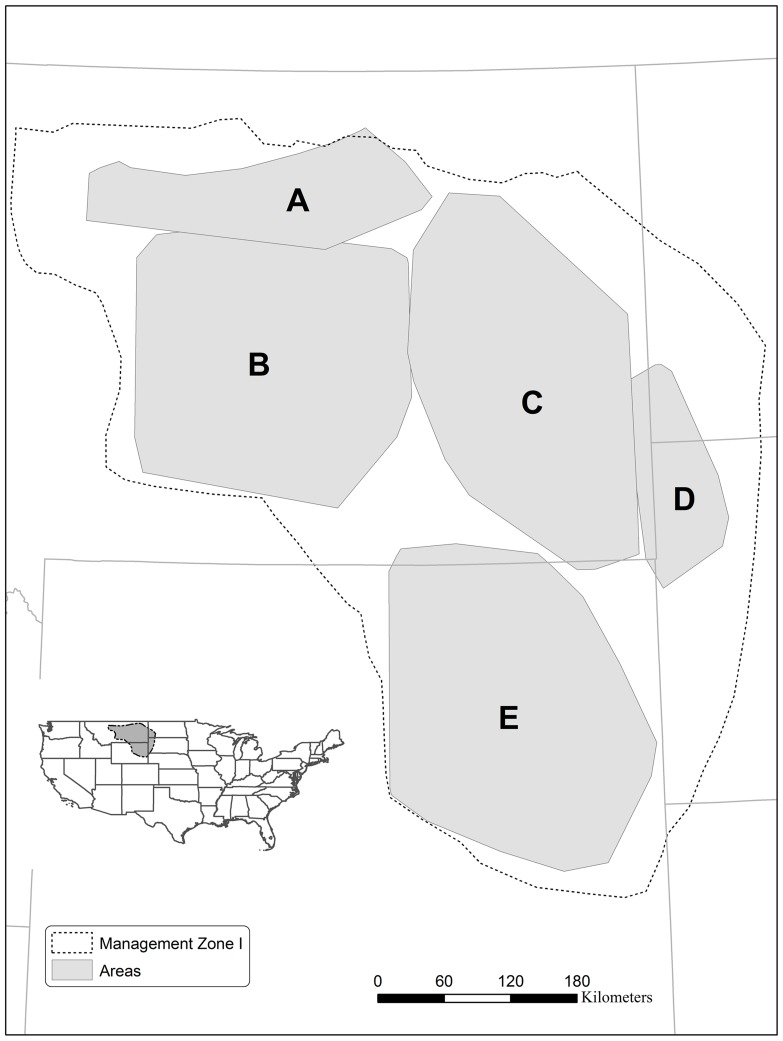
Study region and the sub-areas it contains. A) North-central Montana, B) Central Montana, C) Eastern Montana, D) Western Dakotas, and E) Northeast Wyoming.

While our primary interest was in northeast Wyoming, this area contains such extensive energy development that we widened our study region to encompass less developed and undeveloped areas as well. Our study region covered 20 million ha of eastern Montana, northeast Wyoming and the western Dakotas, encompassing most of the Western Association of Fish and Wildlife Agencies (WAFWA) sage-grouse Management Zone I (Fig. 1). The region is generally dominated by large stands of sagebrush, with an understory of native and nonnative grasses and forbs. Dominant land uses are a mixture of cattle grazing and tillage agriculture, interspersed with concentrated areas of energy development [18]. West Nile virus is omnipresent throughout the region [24].

The tradeoff for having a large-scale study capturing large regional variation in energy development was that detailed, accurate vegetation data were unavailable for such a vast expanse of land. Thus, to capture variation in lek size due to natural landscape attributes within the region, we divided the study region into five sub-areas (Fig. 1), based on the WAFWA sage-grouse subpopulation designations [7]. Sub-areas included our focal area, northeast Wyoming, and the four sub-areas that added varied stressor intensities to our analyses: north-central Montana, central Montana, eastern Montana and western Dakotas.

### Data Sources

We defined a sage-grouse lek as a site where multiple males have been recorded displaying on multiple visits [18]. We obtained lek count and location data from the government agencies responsible for maintaining these data. We censored any leks with errors detected, in conjunction with agency personnel, as well as leks known to be destroyed by housing subdivisions or mining. If a lek was counted multiple times within a year, we used the maximum count for that year [18], [25].

Because leks often occur in a complex, with males moving among leks within 2.5 km of each other, we defined the largest and most regularly attended lek in the group as the complex center [7]. We used the count from each complex center to represent the entire complex, eliminating from the database counts from the smaller and less attended satellite leks. Hereafter, the term ‘lek’ refers to the sample unit of our analyses, which includes complex centers and single leks that were not part of a complex.

For each lek, we used the value of the most recent count that was collected between 2003 and 2009 (last count), excluding from the analyses any leks that became inactive (count = 0) before 2003 and remained inactive on all subsequent counts. We used a single, post-2002 count instead of a time series analysis for two reasons. First, only a small, non-random group of leks have sufficient data to support a time series analysis. Second, we restricted our study to the years after which WNv was first detected in the study region. West Nile virus was first detected in the study region in 2002 [5], but leks are counted in early spring, before the majority of WNv transmission occurs in late summer, thus the effects of the disease could not have been apparent in lek counts until spring, 2003.

Energy development was quantified by the density of producing oil and gas wells near the lek on April 1 in the sample year. We defined the sample year as the year of the last count, both for active leks (last count >0) and for the inactive leks (last count = 0) that had a positive penultimate count. For inactive leks with an unbroken string of zero counts, the sample year was the year of the first zero count. We calculated well density within the following radii of leks to capture a range of potential ecological and management processes relevant to sage-grouse: 1.0 km, 3.2 km, 5 km, 10 km, and 15 km and 20 km. Processes that impact breeding birds at leks should be captured by the 1 km radius [18]. The 3.2 km radius was previously found to be a best-fit scale for detecting energy impacts [18], and it has been used to implement some drilling restrictions [16] and to predict lek losses due to energy development [13]. Telemetry studies in the eastern portion of the sage grouse range (Management Zone 1, Fig. 1) have found that over 95% of nests are within 15 km of the lek at which the female was captured [26], [20]. Finally, effects in our study region have previously been discerned as far as 20 km from development [21]. Because lag times have previously been used in analyses that correlated well density with lek inactivity [17], [18], [19], we initially explored the effect of incorporating a 1–10 year lag time between the sample year and the measured well density. Real time measurements consistently outperformed lagged measurements in the initial analyses, thus we dropped lag times from subsequent analyses.

West Nile virus outbreaks were documented in multiple species in the summers of 2003 and 2007 [5], and outbreaks in sage-grouse populations in Montana, Wyoming, and South Dakota severely impacted survival and nearly extirpated at least one population [14], [15], [24], [27]. Because these outbreaks had the potential to affect spring 2004 and 2008 lek counts, respectively, we assigned positive outbreak year status to each lek whose sample year was 2004 or 2008. Although we refer simply to ‘WNv outbreak years’, we note that other environmental variables may have been associated with those years and may partly explain the population-level effects that occurred during WNv outbreak years.

### Statistical analyses

#### Model Structure

Our analyses were based on lek count regressions. Using lek counts allowed us to estimate the immediate relationship between stressors and the total number of males in a sub-area by simultaneously estimating lek activity and the number of males at active leks. Most lek count analyses have been based on activity-inactivity data, and have thus needed to incorporate lag times to account for the time energy development takes to deplete leks to the point of inactivity (e.g. [17], [18], [19]).

We used a zero-inflated negative binomial (ZINB) likelihood function, which is ideally suited to overdispersed count data, where the variance is a strongly increasing function of the mean, and there are an unusually large number of zero counts [28]. The ZINB is a mixture of a negative binomial distribution and a point mass at zero, meaning that some zero counts are generated by the negative binomial distribution, and some are generated by the point mass of zeros, but all positive counts come from the negative binomial distribution. We parameterized the ZINB so the negative binomial distribution was described by a mean and overdispersion parameter, and the mixing parameter (which governs the amount of zero-inflation) was the probability that a count belonged to the negative binomial distribution. We used a log link for the negative binomial mean and a logit link for the mixing parameter.

We used randomized quantile residuals [29] to validate the fit of the ZINB likelihood function to the data. Unlike most generalized linear model residuals that exhibit only asymptotic normality, randomized quantile residuals are normally distributed, provided the data are distributed according to the specified likelihood function. Thus we used these residuals to verify that the ZINB was an appropriate likelihood function. We used profile likelihood confidence intervals for parameters and 10,000 case-based, nonparametric bootstrap samples to estimate 95% confidence bands for predicted lines. All analyses were conducted in the R programming environment, version 2.10.0 [30].

#### Identifying spatial extent of energy impacts

To identify the spatial scale at which energy impacts to sage-grouse leks are best detected, we repeated, for each radius, a regression of lek count against well density. Each regression used the same well density to predict the negative binomial mean (NB component) and the mixing parameter (ZI component). We compared the resulting six non-nested models with AIC [31]. Because the six models all had the same number of parameters, this procedure was equivalent to comparing the models' maximum likelihood values.

#### Testing for interactions and reducing the model

To test for interactions between energy development and WNv outbreak years, we first fit a saturated model that contained each of the following effects for both the NB and ZI components: well density (measured at the best-fit radius obtained above), a factor for WNv outbreak year, a well density by outbreak year interaction, and a factor for sub-area. The factor for sub-area adjusted for differences in lek size due to natural variation across the study region, allowing for the possibility that leks might be naturally larger or smaller in northeast Wyoming (where most of the high well densities exist) than in other parts of the study region. We tested each interaction term separately using a likelihood ratio test to determine if it was significant (p≤0.05). To obtain a reduced model with which to make predictions, we first removed any non-significant interactions. We then tested the significance of main effects on which significant interactions did not depend, and removed any with p>0.05.

#### Predictions

We used our reduced model to predict the effects of different well densities on lek counts in the northeast Wyoming sub-area, subsequent to a WNv outbreak year, and absent an outbreak year. We make predictions for well densities up to the highest federal permitting level of 3.1 wells/km^2^, which is similar to the maximum well density observed at the 20 km radius (2.9 wells/km^2^), and below the maximum well density at all other radii (3.6–7.9 wells/km^2^).

In particular, we used the estimated parameters to calculate the probability a lek was active, the mean count at active leks, and the total northeast Wyoming lek count (total count). We obtained the total count as the product of the mean count at all leks (whether or not they were active) and the number of leks from northeast Wyoming. We also calculated the number of leks that would be inactive (0 males), as well as the number in small (<11 males), medium (11–25 males) and large (>25 males) size categories [20], by calculating the probability a lek would fall into a category and multiplying it by the number of leks from northeast Wyoming.

## Results

### Identifying spatial extent of energy impacts

When lek count was regressed on well density alone, the 20 km radius explained the variation in the data better than did 4 of the 5 other radii (ΔAIC>2, Table 1). While the 20 km radius provided a nominally better fit than did the 5 km radius, it was statistically indistinguishable (ΔAIC<2). To confirm whether or not the 20 km radius better explained the variation in the data than did the 5 km radius, we compared AIC values for these two radii using the saturated model. The 5 km radius had a ΔAIC value >4 points higher than the 20 km radius, confirming the best fit was achieved using the 20 km radius.

**Table 1:** ΔAIC values from univariate and saturated models used to determine the best fit radius from a lek within which to measure the density of oil and gas wells.

	ΔAIC	
Radius (km)	Univariate	Saturated
20.0	0.00	0.00
5.0	1.44	4.89
15.0	2.09	NA
1.0	4.50	NA
3.2	4.52	NA
10.0	4.78	NA

### Sample sizes of leks subject to different stressors

Our analysis used 1139 leks, 60% of which had producing oil and gas wells within the best-fit 20 km radius, and 40% of which did not (Table 2). Twenty percent of leks were assigned a positive outbreak year status, and 80% were not: this did not depend on the presence or absence of oil and gas wells within 20 km. Leks in different sub-areas were subject to different levels of stressors, underscoring the need for our study region to include sub-areas beyond the northeast Wyoming focal area. The percent of leks within 20 km of oil or gas wells varied from 29% in north-central Montana to 100% in northeast Wyoming, and the percent of leks with positive outbreak year status varied from 11% in the western Dakotas to 27% in eastern Montana.

**Table 2:** Sample sizes of leks in study region categorized by sub-area, presence of wells within the 20 km best-fit radius and whether or not the lek was assigned positive WNv outbreak year status.

			Area					
	WNv?	Wells?	NE WY^1^	NC MT^2^	C MT^3^	E MT^4^	W DK^5^	Category Total
	No	No	1	88	126	144	15	374
	No	Yes	304	35	84	64	57	544
	Yes	No	0	12	25	54	2	93
	Yes	Yes	65	6	27	23	7	128
Area Total			370	141	262	285	81	1,139

1 Northeast Wyoming.

2 North-central Montana.

3 Central Montana.

4 Eastern Montana.

5 Western Dakotas.

### Testing for interactions and reducing the model

Interactions between well density and outbreak year were significant in the ZI (p = 0.0051) and NB (p = 0.0058) components. The coefficient for the ZI interaction was negative, meaning there was more zero-inflation at high well densities in an outbreak year compared to a non-outbreak year. The coefficient for the NB interaction was positive, meaning the negative binomial mean was higher at high well densities in an outbreak year compared to a non-outbreak year. The final model contained all effects from the saturated model (Table 3), including the factors for sub-area (NB component, p = 2×10^−12^; ZI component, p = 2×10^−22^). Because the interactions between well density and outbreak year were significant, we did not test the underlying main effects for removal. Nevertheless, the main effect of well density was seen primarily in the NB component where the distribution's mean decreased with increasing well density. The main effect of outbreak year was seen primarily in the ZI component, where zero-inflation was higher in outbreak years. Both of these main effects are reflected by parameter confidence intervals that do not overlap zero.

**Table 3:** Maximum likelihood estimates and profile likelihood confidence intervals for parameters of the reduced model.

Parameter	Component^1^	MLE	CI
Overdispersion	NB	1.539	(1.354, 1.738)
Intercept	ZI	2.897	(2.240, 3.077)
Intercept	NB	3.352	(3.211, 3.499)
Central Montana	ZI	−1.431	(−2.329, −0.711)
Central Montana	NB	−0.413	(−0.600, −0.228)
Eastern Montana	ZI	−1.047	(−1.949, −0.305)
Eastern Montana	NB	−0.809	(−0.997, −0.624)
Western Dakotas	ZI	−0.652	(−1.778, 0.618)
Western Dakotas	NB	−1.023	(−1.277, −0.764)
Northeast Wyoming	ZI	−2.135	(−3.044, −1.402)
Northeast Wyoming	NB	−0.463	(−0.672, −0.254)
Well Density	ZI	0.269	(−0.079, 0.656)
Well Density	NB	−0.369	(−0.505, −0.230)
Outbreak Year	ZI	−1.328	(−1.732, −0.930)
Outbreak Year	NB	−0.168	(−0.351, 0.019)
Well*Outbreak Year Interaction	ZI	−1.406	(−2.751, −0.380)
Well*Outbreak Year Interaction	NB	0.765	(0.199, 1.514)

1 Parameters belonging to the negative binomial (NB) model component are presented on the log scale. Parameters belonging to the zero-inflation (ZI) model component (mixing parameter) are presented on the logit scale. The mixing parameter was defined as the probability that a count belonged to the negative binomial distribution.

### Predictions

#### Effect of Well Density in the Absence of an Outbreak Year

**Figure 2:**
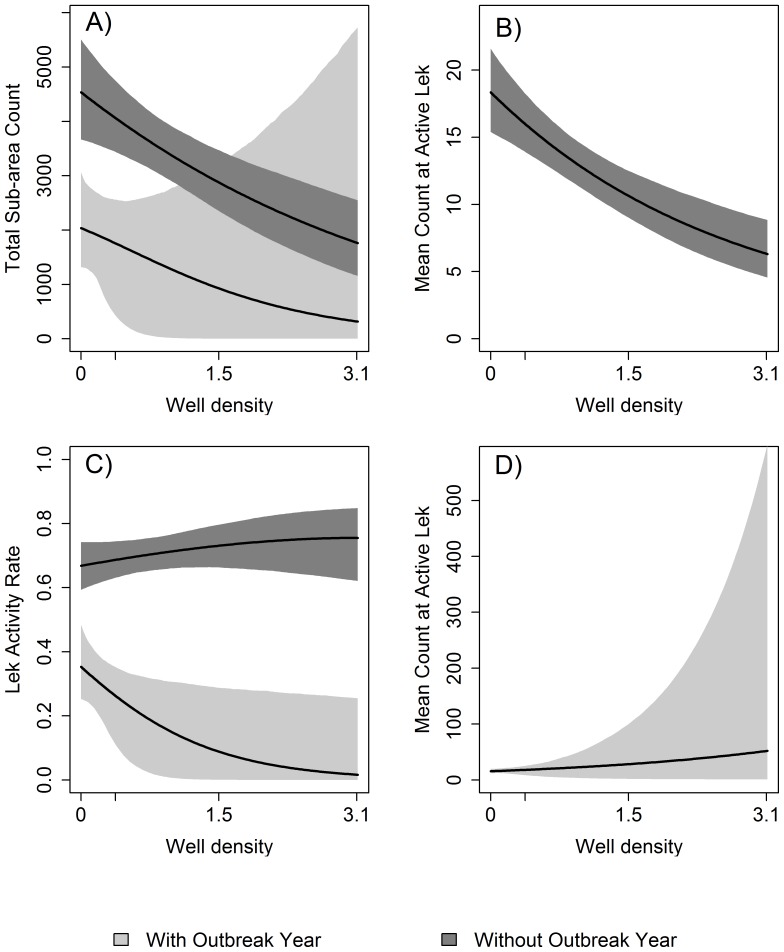
Predictions for Northeast Wyoming sage-grouse. Lines show expected values, and shaded areas are 95% confidence regions. Oil and gas well density is given in # wells/km^2^ within a 20 km radius of each lek. A) Total count of male sage-grouse on all leks in northeast Wyoming, B) Mean count of male sage-grouse at active leks absent a WNv outbreak year, C) Proportion of leks that are active, and D) Mean count of male sage-grouse at active leks subsequent to a WNv outbreak year. Note difference in scale of y-axis between B and D.

Absent an outbreak-year, drilling an undeveloped landscape to the permitting level of 3.1 wells/km^2^ reduced the predicted total northeast Wyoming count (total count) by 61% (Fig. 2A, Table 4), from an estimated 4537 males to 1768 males. Underscoring the strength of this reduction are the non-overlapping 95% confidence intervals [CI: 3668, 5507] and [CI: 1162, 2554]. The decrease in the total count was caused by the decrease in the mean count at active leks, which declined from 18 [CI: 15, 22] to 6 [CI: 5, 9] (Fig. 2B). Furthermore, the decreasing mean count at active leks resulted from fewer large and medium-sized leks and more small leks (Table 4). For example, the 60 [CI: 42, 80] large leks predicted to exist if northeast Wyoming had no oil and gas development decreased to 2 [CI: 0,10] with development to 3.1 wells/km^2^. Conversely, the 91 [CI: 73, 111] small leks predicted to exist without development increased to 232 [CI: 180, 266] at 3.1 wells/km^2^. Lek inactivity rates, however, remained relatively constant between and 24% [CI: 15%, 38%] and 33% [CI: 26%, 41%], regardless of well density (Fig. 2C).

**Table 4:** Predicted total lek count and number of inactive (0 males), small (1–10 males), medium-sized (11–25 males) and large (>25 males) leks for northeast Wyoming as a function of oil and gas well density (#/km^2^) within 20 km of each lek and WNv outbreak or non-outbreak year status.

Without West Nile Virus Outbreak Year										
			Number of Leks							
Well Density	Total Lek Count		Inactive		Small		Medium-sized		Large	
	Mean	95% CI	Mean	95% CI	Mean	95% CI	Mean	95% CI	Mean	95% CI
0.00^3^	4537	(3668, 5507)	123	(95, 151)	91	(73, 111)	96	(84, 108)	60	(42, 80)
0.39^3^	4062	(3439, 4753)	116	(94, 136)	108	(91, 125)	98	(89, 108)	48	(34, 62)
0.75^1^	3648	(3147, 4204)	110	(91, 129)	125	(109, 142)	99	(90, 108)	37	(26, 49)
1.54^3^	2876	(2352, 3471)	100	(75, 125)	163	(138, 190)	89	(74, 103)	18	(10, 29)
2.87^2^	1895	(1288, 2670)	91	(57, 137)	224	(175, 259)	52	(25, 84)	3	(0, 12)
3.09^3^	1768	(1162, 2554)	91	(56, 140)	232	(180, 266)	46	(19, 80)	2	(0, 10)

With West Nile Virus Outbreak Year										
			Number of Leks							
	Total Lek Count		Inactive		Small		Medium-sized		Large	
Well Density	Mean	95% CI	Mean	95% CI	Mean	95% CI	Mean	95% CI	Mean	95% CI
0.00^3^	2037	(1318, 3062)	239	(191, 277)	57	(38, 82)	51	(36, 69)	23	(11, 41)
0.39^3^	1757	(430, 2558)	273	(240, 329)	36	(13, 53)	38	(11, 51)	23	(2, 37)
0.75^1^	1473	(65, 2616)	299	(252, 361)	23	(2, 39)	27	(1, 45)	21	(0, 39)
1.54^3^	927	(1, 3212)	337	(263, 370)	7	(0, 23)	11	(0, 35)	14	(0, 50)
2.87^2^	373	(0, 5246)	362	(274, 370)	1	(0, 11)	2	(0, 21)	5	(0, 67)
3.09^3^	319	(0, 5712)	364	(275, 370)	1	(0, 9)	1	(0, 19)	4	(0, 68)

1 Mean well density in 2009 within a 20 km radius of northeast Wyoming leks.

2 Maximum well density in 2009 within a 20 km radius of northeast Wyoming leks.

3 Permitting levels for well density on United States federal land.

#### Effect of Outbreak Year in the Absence of Oil and Gas Development

In the absence of energy development, we predicted a WNv outbreak year would cause the total count to decline by 55% (4537 [CI: 3668, 5507] to 2037 [CI: 1318, 3062]), which is similar to the 61% reduction achieved by drilling to 3.1 wells/km^2^. The outbreak year reduction in the total count was driven by a near doubling of lek inactivity, from 33% (26%, 41%) to 65% (51%, 75%). The mean count at active leks remained relatively constant between 16 [CI: 12, 20] and 18 [CI: 15, 22] males.

#### Combined Effects of Well Density and Outbreak Year

The mean inactivity rate associated with outbreak years increased with increasing energy development, but with high variation around predictions. The 65% [CI: 51%, 75%] inactivity rate predicted without development rose to 98% [CI: 74%, 100%] with development to 3.1 wells/km^2^.

While the mean count at active leks subsequent to an outbreak year increased with increasing energy development, this result was unreliable. Unrealistically large confidence intervals for active leks with high well densities and positive outbreak year status precluded meaningful predictions for their mean count (Fig. 2D). With an outbreak year and no oil and gas development, the mean count at active leks was 18 [CI: 15, 22]. With an outbreak year and 3.1 wells/km^2^, the confidence interval had a lower limit of 1 and an upper limit of 594 males, compared to a maximum lek count of 115 males in the raw data. This uncertainty is a direct result of how few active leks in areas of high oil and gas development were last counted after an outbreak year. In particular, of the 1139 leks in our analysis, only 2 that were last counted subsequent to a WNv outbreak year were also active and had well densities >1.5 wells/km^2^ in the 20 km radius.

The uncertainty in the mean count at active leks with high well densities and an outbreak year is also reflected in the expected total count under these conditions. However, at lower well densities, the relationship between well density, outbreak year and total count is less variable. For example, at 0.75 wells/km^2^, we predicted an outbreak year would produce a total count of 1473 [CI: 65, 2616] males, 40% of the 3648 [CI: 3147, 4204] males predicted at the same well density subsequent to non-outbreak year.

## Discussion

Energy development and WNv are both outside the sage-grouse evolutionary experience, and our work is the first to quantify their combined effects, across a 20 million ha region encompassing a wide range of stressor intensities. Combinations of stressors are important drivers of extinction, particularly when they are outside the evolutionary experience of a species [2]. The doubling of lek inactivity rates as a result of outbreak years is consistent with the extreme susceptibility of sage-grouse to this novel disease [32] and near population extirpation observed in the field [15]. Furthermore, our work shows the progression of effects on lek counts previously documented as a 2–10 year time lag between the onset of development and lek inactivity [17], [18], [19]. We demonstrate an immediate decrease in lek size due to increasing well density, increased inactivity subsequent to outbreak years, and an inactivity rate subsequent to outbreak years that is worsened by high levels of oil and gas development.

Multiple mechanisms may underlie the synergistic effects of energy development and outbreak years on lek inactivity. First, the prevalence of WNv may be higher within coal bed natural gas fields [14], [18] because ponds created from ground water brought to the surface during gas extraction provide additional habitat for the mosquito, *Culex tarsalis*, a known WNv vector [15], [33], [34], [35]. On the other hand, increasing inactivity of small leks after outbreak years could have resulted from other unfavorable environmental conditions associated with the outbreak years in our analyses. For example, higher summer temperatures can increase WNv transmission [14], but high temperatures can also be associated with drought, which can affect range conditions, and as a result, chick production [36]. We suggest that reductions in the size of active leks as a result of energy development may set the stage for other stressors to cause lek inactivity, because sage-grouse populations in developed areas are already small, and vulnerable to stochastic events [37]. While WNv is a major threat to sage-grouse in the eastern portion of their range, and one whose outbreaks correlate well with lek inactivity, it is but one threat to populations already reduced by energy development. In other areas, events such as wildfire are as much or more of a threat to sage-grouse than WNv [38], and they, too, might become the final straw for populations reduced by energy development. Moreover, species like sage-grouse, whose productivity and population growth rate vary markedly from year to year [39], may be particularly vulnerable to extirpation from even a single stressor. The high variation in population growth guarantees episodic years in which the species fares poorly, thus reduced population size due to one stressor may be exacerbated to the point of extirpation as a result of natural fluctuations in population growth.

The scale at which sage-grouse are impacted by energy development now appears to be larger than initially thought. We suggest that sage-grouse may respond to energy development primarily at a large scale, and secondarily in close proximity to the lek. The primary, large-scale response of sage-grouse to energy development is supported both by our work and by previous studies that have found similarly large scales of impact in mostly undeveloped regions [20] or in regions that contained a mixture of highly developed and undeveloped areas [21]. The secondary, local response is supported by the comparatively small scales of impact in localities already developed for oil and gas extraction [17], [18], [19], [40], most of which were highly developed by the time of study [17], [18], [19]. If much of an area is already developed, effects at impacted sites may appear artificially small because the nominal control areas may also be affected by development [22]. Future research should further evaluate the possibility of oil and gas development having both large and small scale effects on sage-grouse populations.

**Figure 3:**
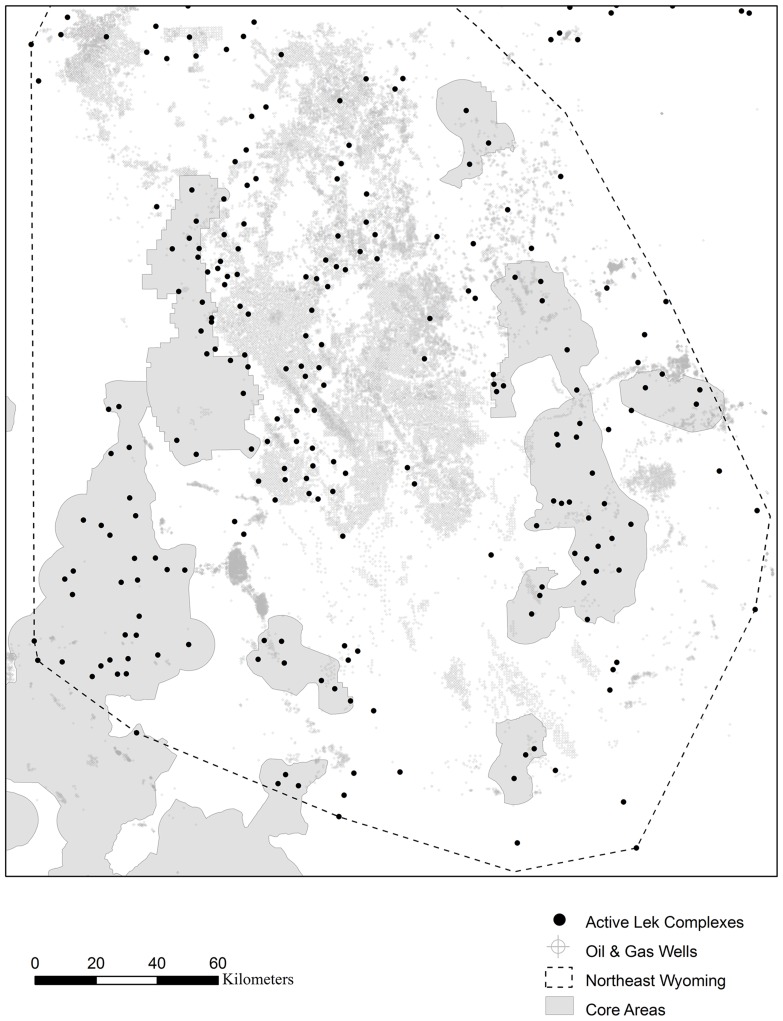
Northeast Wyoming leks, oil and gas wells and core sage-grouse management areas. As of 2009, the pictured leks were active and the pictured wells were producing.

A persistent problem in the conservation of declining populations is a disparity between the spatial scale of management actions and the scale at which populations respond [3]. When we account for the large-scale response of sage-grouse to energy development, the effective size of core conservation areas designed to protect sage-grouse populations in northeast Wyoming becomes negligible. In fact, as of 2009, all active leks in northeast Wyoming had at least one producing oil and gas well within 20 km. This results from widespread oil and gas development in northeast Wyoming, its core areas having been selected after substantial energy development had already occurred, and boundaries having been delineated to include leks, but not necessarily a buffer between the lek and the development boundary (Fig. 3). While a lek provides an important center of breeding activity, and a conspicuous location at which to count birds, its size is merely an index to the population dynamics in the surrounding habitat. For example, in the eastern portion of their range (Management Zone 1), female sage-grouse captured on a lek use an approximately 15-km radius around the lek for nesting; by contrast, a 3.2-km radius (which is used for some drilling restrictions near leks) encompasses only 35–50% of nests associated with the lek [26], [20]. Thus attempting to protect a lek, without protecting the surrounding habitat, provides little protection at all.

Coupling our findings with those of others [6] further characterizes sage-grouse as a wildland species that requires large, intact sagebrush landscapes, and is largely intolerant of human disturbance. Our work compliments new science from the western portion of the species' range where active leks have only 0.3% developed land within a 5 km radius compared to 8.7% at inactive leks [41]. To deliver conservation more effectively, each of 11 western sage-grouse states recently delineated core areas for conservation [42], and most policies restrict or eliminate new developments inside these areas of high bird abundance. Core area policy in Wyoming, for example, restricts new development to an average of one oil or gas well pad per 2.6 km^2^, places a 5% cap on total disturbance, and steers new drilling to less impactful places [16]. While abatement measures for WNv are sensible [14], placing new developments outside of core areas has the greatest likelihood of sustaining populations. The 2012 Near-Term Greater Sage-Grouse Conservation Action Plan lays out a spatially explicit approach by identifying the threats for each core area, the appropriate conservation action to abate those threats, and the probability of success if those actions are fully implemented [43]. Our findings corroborate this spatial approach for reducing all stressors in core areas so that beneficial conservation measures are not negated by detrimental actions.
